# Mesenchymal Stem Cells Exhibit Both a Proinflammatory and Anti-Inflammatory Effect on Saccular Aneurysm Formation in a Rabbit Model

**DOI:** 10.1155/2019/3618217

**Published:** 2019-07-22

**Authors:** Michael B. Avery, Brooke L. Belanger, Amy Bromley, Arindom Sen, Alim P. Mitha

**Affiliations:** ^1^Department of Neurosciences, University of Calgary, Alberta, Canada; ^2^Neuroscience Graduate Program, University of Calgary, Alberta, Canada; ^3^Department of Pathology and Laboratory Medicine, University of Calgary, Alberta, Canada; ^4^Pharmaceutical Production Research Facility, Schulich School of Engineering, University of Calgary, Alberta, Canada; ^5^Hotchkiss Brain Institute, University of Calgary, Alberta, Canada

## Abstract

Several studies have demonstrated a potential interaction between mesenchymal stem cells (MSCs) and saccular aneurysms. In this study, we sought to determine whether allogenic bone marrow-derived MSCs had the ability to prevent aneurysm formation in a known rabbit elastase aneurysm model. MSCs were injected intravenously in experimental rabbits at the time of surgical creation and two weeks postcreation and compared with control rabbits receiving vehicle injection. Angiography was used to compare aneurysm measurements four weeks postcreation, and aneurysms were harvested for histological properties. Serum was collected longitudinally to evaluate cytokine alterations. Serum from control animals was also utilized to perform *in vitro* tests with MSCs to compare the effect of the serologic environment in animals with and without aneurysms on MSC proliferation and cytokine production. While aneurysm morphometric comparisons revealed no differences, significant cytokine alterations were observed *in vitro* and *in vivo*, suggesting both anti-inflammatory and proinflammatory processes were occurring in the presence of MSCs. Histological analyses suggested that tunica intima hyperplasia was inhibited in the presence of MSCs.

## 1. Introduction

It is estimated that approximately 3% of the general population harbors and intracranial saccular aneurysm, a pathological dilation of the cerebral arteries at bifurcation points that is prone to rupturing [1]. Aneurysmal rupture occurs in 10 of every 100,000 people annually and carries with it an estimated mortality rate of 50% and a significant neurological disability rate among survivors of approximately 30% [2]. Several key changes in the arterial wall occur that are thought to lead to aneurysm formation, including loss of elastin content in the internal elastic lamina and reduction of the tunica media leading to weakening of the wall [3]. Pulsatile blood pressure forces ensue, leading to outpouching of the thinned wall. Various inflammatory cascades have been shown to mediate these processes, with alterations of wall shear stresses and wall shear stress gradients from hypertension creating endothelial dysfunction alongside predisposing patient factors such as smoking [4, 5].

While there are several good treatment options available for cerebral aneurysms, there is as of yet no viable therapeutic option to minimize or prevent the initiation or progression of their pathogenesis. Many prevention strategies have aimed at disrupting the implicated inflammatory cascades. Unfortunately, they have been fraught with limited success or impossible human application and have largely been focused on abdominal aortic fusiform aneurysms [6–14]. There is evidence to suggest that saccular and fusiform aneurysms exhibit sufficiently distinct pathogenetic processes, warranting investigations into saccular aneurysm models, as it is unlikely that prevention strategies will be translatable between the two aneurysmal pathologies [15].

Mesenchymal stem cells have been studied as a way to modulate inflammatory processes and interrupt pathogenesis for a variety of different diseases. They are immune-evasive, making them an intriguing approach for human disease models [16–18]. The immunomodulatory properties of MSCs can vary based on their environment and have thus been investigated as an adjuvant therapy during the endovascular treatment of cerebral saccular aneurysms [19]. Studies by our laboratory and others have suggested that MSCs introduced at the time of treatment of saccular aneurysms in animal models have the capacity to improve aneurysm histological healing [20–22]. However, no studies have been performed to investigate the effects of MSCs on the pathogenesis of saccular aneurysms. We hypothesized that MSCs introduced intravenously (IV) during the formative processes of saccular aneurysms in a rabbit elastase model would inhibit the development of aneurysms through the anti-inflammatory properties of MSCs.

Characterization is required to positively identify cells as bone marrow-derived MSCs. After extraction from the tibia, tests include the expression of surface markers CD44, CD105, CD29, and negative for HLA-DR. Differentiation abilities into osteogenic, chondrogenic, and adipogenic lineages were also analyzed. A protocol has been derived in our lab to carry out these experiments, and cells in the following experiments were taken from an established cell line.

## 2. Materials and Methods

This study received ethics certification from our institution's animal care committee (study identification number AC16-0060). All procedures were in accordance with institutional guidelines.

### 2.1. Study Design

Twenty-six New Zealand White Rabbits were assigned 1 : 1 in an alternating fashion to an experimental group and a control group. Animal caretakers, histologists, and pathologists, but not surgeons or angiographers, were blinded to the allocations. For each rabbit, saccular aneurysms were surgically created in the proximal right common carotid arteries on day 1, with 5 cc of arterial blood drawn. During the procedure (day 1), control rabbits received an IV injection of 2 mL of PBS (Life Technologies, Cat. No. 10010-023), while experimental rabbits received an IV injection of 5 × 10^6^ MSCs suspended in 2 mL of PBS, both followed with a 2 mL PBS flush. The dose of MSCs selected was in keeping with doses reported in previous studies [20, 21]. Identical injections were also administered on day 15. On days 1, 15, and 29, 5 cc arterial blood draws occurred in all rabbits for serum analysis. Rabbits underwent digital subtraction angiography (DSA) on day 29 to visualize and measure the saccular aneurysm and were terminated afterwards. Aneurysms were surgically harvested for histological analysis.

Primary outcome was aneurysm greatest dimension, in any orientation, as measured on DSA on day 29. Secondary outcomes included other angiographic measurements including greatest dome length; greatest dome thickness and dome volume; serum cytokine and MMP levels at days 1, 15, and 29; and histological analyses including elastin semiquantification analysis of aneurysm dome walls, aneurysm wall thickness, and tunica intima thickness.

### 2.2. MSC Culture

Bone marrow-derived MSCs were thawed from a pool of purified New Zealand White Rabbit MSCs. Cells in this pool were initially isolated, characterized as MSCs, and cultured in serum-free medium, PPRF-msc6, developed in our laboratory [23]. Thawed cells were again cultured in PPRF-msc6 for 72 hours, followed by a passage using TrypLE Express (ThermoFisher Scientific, Cat. No. 12605028) with a plating density of 5,000 MSCs/cm^2^ for another 72 hours of culture growth time. MSCs were then lifted again and counted, assimilating cultures as needed to obtain 5 × 10^6^ MSCs for each use. Cells were washed and suspended in 2 mL of PBS (Life Technologies, Cat. No. 10010-023) and transferred to a 3 mL syringe, capped with a 20 Ga needle. Suspended cells were used within 30 minutes.

### 2.3. Saccular Aneurysm Creation

Each of twenty female New Zealand White Rabbits (Charles River Laboratories International, Canada), aged 13 weeks, had a right common carotid artery aneurysm surgically created using the elastase-induced saccular aneurysm model described by Hoh et al. [24]. To summarize, animals were anesthetized using acepromazine 0.15 mg/kg IV and intubated. Anesthesia was maintained with inhaled 5% isoflurane in 100% O_2_ at 3 L/min. Enrofloxacin was administered SC at 10 mg/kg, followed by ketoprofen 1 mg/kg SC and buprenorphine 0.03 mg/kg SC for analgesia. The right central ear artery was used to obtain 5 mL of arterial blood in a serum separation tube, which was spun in a centrifuge at 1000×g for 10 minutes at 4°C. Serum was collected and stored immediately at -80°C.

Under sterile conditions, a right paramedian vertical neck incision was made and the right common carotid artery was identified and dissected to its origin. Rabbits received an IV injection of either 2 mL of PBS or 5 × 10^6^ MSCs suspended in 2 mL PBS based on group allocation, followed by a 2 mL PBS flush. A temporary aneurysm clip was placed at the origin, and the artery was ligated 3 cm distal. The artery was cannulated in a retrograde fashion 2.5 cm distal to the clip with an angiocatheter, and approximately 35 units of elastase (Worthington Biochemical Corporation, Cat. No. LS002279) was infused into the lumen. The angiocatheter entry site was secured with a silk tie. After twenty minutes, the angiocatheter was removed and the entry site silk tie was knotted tightly. The temporary clip was removed to reestablish blood flow into the blind-ended right common carotid artery. The skin was sutured and the rabbit awoken from anesthetic. Rabbits were observed for four weeks, receiving food and water *ad libitum* with a 12-hour light/dark cycle in our institution's animal care facility. Postoperative antibiotics and analgesia were administered for three days and 36 hours, respectively, at the same doses listed above.

Two weeks postaneurysm creation, all rabbits received a second IV injection of either vehicle or 5×10^6^ MSCs as above. Five mL of arterial blood was again obtained from the right central ear artery of each rabbit. Serum was isolated and stored as above. A final arterial draw and serum collection was performed four weeks postaneurysm creation.

### 2.4. Digital Subtraction Angiography

A digital subtraction angiogram was obtained four weeks postaneurysm creation in all rabbits. While anesthetized and intubated as above, the right femoral artery was surgically accessed and cannulated with a Prelude® 4Fr introducer sheath (Merit Medical, UT). An Impress® 4Fr guide catheter (Merit Medical, UT) containing a 0.035 in Glidewire® guidewire (Terumo Corporation, Japan) was passed into the sheath. Under fluoroscopic guidance of an OEC 9800 C-arm (General Electric, MA), the catheter and guidewire system was advanced to the proximal brachiocephalic artery and parked just proximal to the origin of the right common carotid artery aneurysm. The guidewire was removed, and nonionic iodinated low-osmolar contrast was rapidly injected through the catheter to obtain images of the aneurysm with a right anterior oblique view. A coin measuring 18 mm in diameter was placed in the imaging field for reference to control for the distance between the rabbit and the detector. Maximal dome width, maximal dome length, and greatest dimension (in any orientation) were measured in all rabbits. Aneurysm domes were all elliptical in shape, and so the formula for the volume of an ellipse was used to calculate volume, assuming the 2 short axis lengths were equal due to single plane acquisition.

### 2.5. Serum Samples

Of the 5 mL of arterial blood, one cc of serum was isolated from each rabbit after centrifugation, immediately preaneurysm creation, two weeks postaneurysm creation, and four weeks postaneurysm creation as described above. All serum samples were stored at -80°C within 30 minutes of acquisition. Processing of all samples occurred simultaneously after acquiring all samples for the study. The serum cytokine levels evaluated in the serum samples included interleukin 1*β* (IL-1*β*), interleukin 10 (IL-10), tumor necrosis factor *α* (TNF-*α*), macrophage inflammatory proteins 1*α* and 2 (MIP-1*α* and MIP-2, respectively), monocyte chemoattractant protein 1 (MCP-1), and vascular endothelial growth factor (VEGF). Cytokines were quantified using a Mouse Cytokine Array/Chemokine Array 32-Plex (Eve Technologies Corporation, Canada) antibody array kit. Matrix metalloproteinase 2 (MMP-2) and proMMP-9 were quantified as well using a Mouse MMP Discovery Array 5-Plex (Eve Technologies Corporation, Canada) antibody array kit. No rabbit arrays were available, and preliminary replicated testing with rabbit serum produced results with reasonable precision. For both assays, a Bio-Plex™ 200 system (Bio-Rad Laboratories Incorporated, CA) suspension array system was used. For the cytokine assay, a Milliplex Mouse Cytokine/Chemokine kit (Millipore, MO) was used by Eve Technologies according to their protocol, while both a Milliplex Mouse MMP panel 1 kit and a Milliplex Mouse MMP panel 2 kit (Millipore, MO) were used by Eve Technologies according to their protocols for the MMP assay.

### 2.6. Termination and Aneurysm Harvesting

After completion of the day 29 DSA and collection of arterial blood serum sample, rabbits were deeply sedated with 5% inhaled isoflurane, then euthanized via intracardiac injection of 4 mL of formalin in accordance with ethical standards. After approximately 15 minutes, the neck incision was reopened and the aneurysm complex was exposed. A sternotomy was also performed, allowing easy access to the aortic arch. The brachiocephalic artery was identified and followed to the origin of the aneurysm. The aneurysm complex, including a segment of the proximal brachiocephalic artery and distal right subclavian artery, was harvested intact. The total time from euthanization to depressurization of the aneurysm complex via resection took between 30 and 60 minutes, allowing the formalin to penetrate the vessel and aneurysm walls entirely, assuming a penetration rate of 1 mm/hr in the first hour after administration.

### 2.7. Histological Analysis

Harvested aneurysms were immediately placed in formalin for at least five days prior to sequential alcohol dehydration and embedding in paraffin wax blocks. Tissue blocks were sectioned in 5 *μ*m slices and mounted with Verhoeff-van Gieson (VVG) stain to identify the elastin content and boundaries of the three vascular layers. Using optical microscopy, a pathologist (author AB) blinded to group allocation randomly selected five representative regions from each aneurysm and measured the thickness of the tunic media and tunica intima, noting the presence of tunica intima hyperplasia, if present. Tunica media thickness was divided by the tunica media thickness of the parent vessel to normalize the data.

Midaneurysm slices were digitized to semiquantify elastin content in the aneurysm dome wall. A colorimetric analysis tool in Aperio ImageScope (Leica Biosystems, Wetzlar, Germany) was utilized. Using the analytical pen tool, the tunica media was selected, taking care not to include areas of tangential wall cuts or tissue folds that could falsely represent elastin content. A positive pixel algorithm was applied to selected regions that detected black pixels from the VVG stain, representing elastin. The analysis reported the proportion of the selected area containing black pixels.

### 2.8. In Vitro Analysis

To determine whether the intravascular serum environment in the setting of an actively developing aneurysm has an effect on the activity of MSCs, an *in vitro* analysis was performed using the frozen serum from control rabbits that had undergone aneurysm creation without MSC treatment. This experiment is aimed at measuring differences in MSC proliferation and in cytokine levels when introduced to the serum collected from animals without an aneurysm versus those harboring an aneurysm.

MSCs from the previously utilized pool were thawed as above, cultured with serum-free PPRF-msc6 media, and separated into two primary groups. Cells were first thawed and cultured at passage two. Serum samples taken from rabbits above were kept at -80°C until needed, at which point they were thawed in a water bath at 37°C. The first group of cells was cultured in media supplemented with 0.30 mL of serum from MSC-naïve rabbits, collected 29 days after aneurysm creation, thereby simulating an aneurysmal environment. The second group was cultured in PPRF-msc6 media supplemented with 0.30 mL of serum from MSC-naïve rabbits obtained prior to aneurysm creation in order to simulate an aneurysm-free environment. Cells were seeded at a density of 40,000 cells per well in bovine gelatin-coated 6-well plates and incubated for 72 hours. At passage four, cells were counted with a hemocytometer, and media samples were sent to be analyzed by the same cytokine panels from the above experiments. Cytokines were chosen on the basis of the most common cytokines found in the body in relation to inflammation; a panel of 42 total cytokines was utilized. Panel results for these two groups were then compared to the cytokine panel obtained from the serum of MSC-naïve rabbits prior to surgical aneurysm creation.

### 2.9. Statistical Tests

Angiographic outcomes were all tested for normality using a Shapiro-Wilk test and homogeneity of variances with Levene's test. For those data comparisons that were normally distributed with homogeneous variances, groups were compared using an independent sample *t* test. Other data were compared with a Mann-Whitney *U* test.

*In vivo* serum cytokine and MMP levels were not normally distributed; therefore, nonparametric analyses were performed. A within-subject analysis was conducted to compare serum samples over time within each group individually using Friedman's test. Any tests that rejected the null hypothesis were further tested with a post hoc Wilcoxon Signed-Rank test with a Bonferroni correction applied. Next, a between-subject analysis was carried out to directly compare the two groups at each specific time point by using Mann-Whitney *U* tests. *In vitro* serum cytokine and MMP levels were analyzed with Student's *T*-test and Levene's test.

Histological outcomes were first tested for normality, then compared using either a *t* test or a Mann-Whitney *U* test as appropriate. Angiographic and histological measurements used for correlation analyses were normally distributed; therefore, a Pearson's correlation was performed on the data of interest.

All statistical tests were conducted using SPSS Version 21 (IBM, Armonk, NY).

## 3. Results

Twenty-six rabbits were required to complete 20 successful experiments. Five rabbits died intraoperatively. Two were due to anesthetic complications. One developed a pneumothorax with severe hypotension and was euthanized. Surgical complications occurred in the remaining two rabbits with one having an iatrogenic carotid artery perforation and the other developing a spontaneous carotid artery bleed thought to be secondary to excessive elastase spillage onto the external arterial surface throughout the incubation period. An additional rabbit was excluded from the analysis as a large air bubble was found in the proximal common carotid artery after elastase introduction, preventing complete luminal elastase exposure. No postoperative complications occurred in the 20 included rabbits.

### 3.1. Angiographic Outcomes

**Figure 1:**
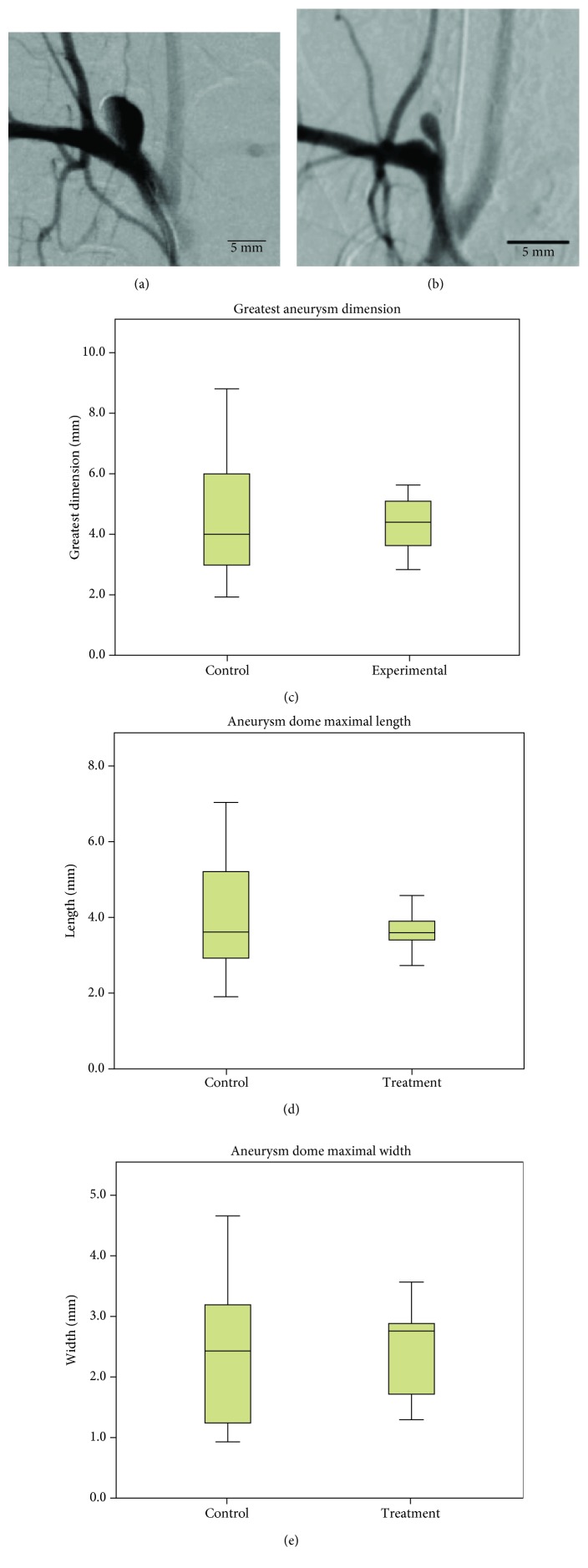
Anteroposterior cerebral angiography; brachiocephalic injection. (a) Largest saccular aneurysm formed in the control group. (b) Largest saccular aneurysm formed in the experimental group. Box and whisker graphs of (c) greatest dimensions, (d) maximal saccular dome length, and (e) maximal saccular dome width of right common carotid artery saccular aneurysms as measured on cerebral angiography. Median, quartiles, and maximum/minimum values shown.

Twenty rabbits formed aneurysms and DSAs occurred without issue. There were no aneurysm ruptures. All aneurysms were elliptical in shape, with one control aneurysm having a small lobule at the distal aspect of the dome. In all but one aneurysm, greatest dimension was equivalent to the greatest length measurement. Control aneurysm mean greatest dimension was 4.76 mm (*s* = 1.59, 95% CI: 3.38 mm – 6.14 mm), while the experimental group had a mean greatest dimension of 4.29 mm (*s* = 0.54, 95% CI: 3.37 mm – 4.84 mm). There was heterogeneity of variances; thus, a Mann-Whitney *U* test was used to conclude that there was no significant difference in greatest dimension (*U* = 44, *p* = 0.65). Greatest dome length and width were statistically equivalent between the control and experimental groups at 3.97 mm, 2.37 mm and 3.59 mm, 2.44 mm, respectively (*U* = 48, *p* = 0.88; *t*(18) = −0.159, *p* = 0.875, respectively; Figure 1).

### 3.2. Histological Outcomes

**Figure 2:**
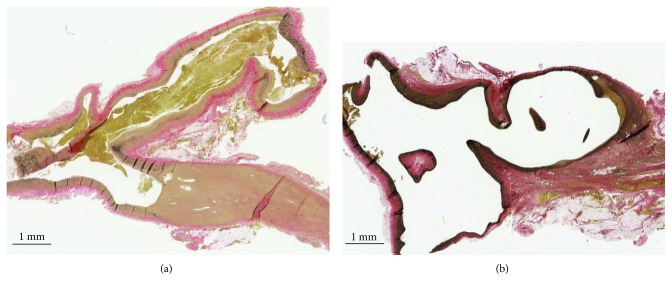
Histological sections of two saccular aneurysms, stained with VVG. Aneurysm sac is seen pointing towards the top right of both slices. The vessel wall consists of tunica adventitia (red), elastin fibers (black) in the tunica media, and a very thin layer of tunica intima adjacent to the lumen. (a) Blood clot is seen filling the aneurysm lumen. (b) Very small amount of blood clot seen at the apex of the aneurysm.

Tissue blocks were obtained for all aneurysms except for one control rabbit aneurysm, which had unrecognizable morphology upon sectioning, precluding accurate aneurysm measurements from being obtained and therefore was unable to be included in the analysis. Varying degrees of blood clots were noted in the domes of all but three aneurysms (Figure 2). The angiograms of these aneurysms did not demonstrate any flow-limiting thrombus; thus, it was concluded that these occurred post-mortem. Histological measurements did not incorporate these thrombi.

**Figure 3:**
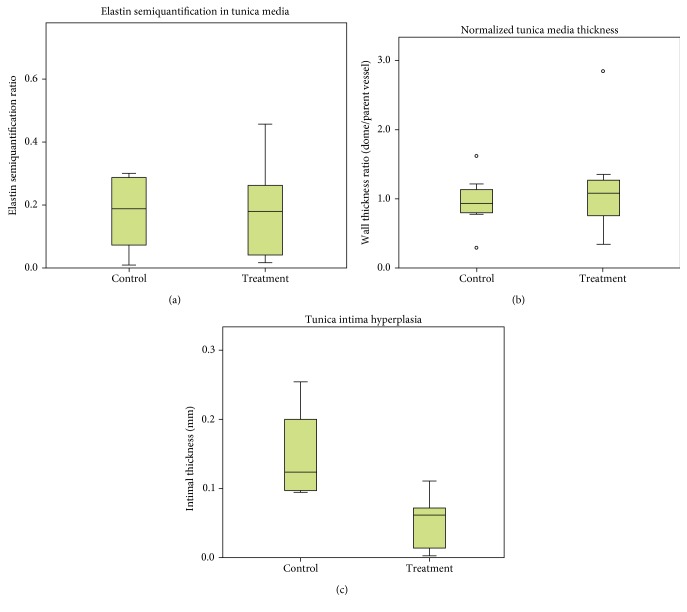
(a) Elastin semiquantification of the tunica media in the control and experimental group aneurysms. No significant difference in elastin content was found (*n* = 9/10, *t* [17] = 0.382, *p* = 0.707). Box and whisker plots demonstrate median, quartiles and maximum/minimum values (withholding outlier, represented by an open circle). (b) Tunica media thickness and (c) tunica intima hyperplasia (when present) measured histologically in the control and treatment group aneurysms. Tunica media thickness was normalized to parent vessel thickness. No significant differences were seen in tunica media thickness (*n* = 9/10, *U* = 37, *p* = 0.514), but the control group demonstrated significantly more tunica intima hyperplasia than the treatment group (*n* = 4/6, *t* [8] = 2.669, *p* = 0.028).

Mean elastin semiquantification ratios for the control group and experimental group were both normally distributed, with means of 0.2116 and 0.1818, respectively (Figure 3). No significant difference was found (*t*(17) = 0.382, *p* = 0.707). Mean dome tunica media thickness was not normally distributed for the experimental group. The control group mean of 0.14 mm was not significantly different from the experimental group mean of 0.157 mm (*U* = 34.5, *p* = 0.391; Figure 3). Intimal hyperplasia was found in 4 of 9 control aneurysms and 6 of 10 experimental aneurysms. When present, tunica intima thickness was measured. Within these groups, the mean control group demonstrated significantly more hyperplasia than the experimental group (0.149 mm vs. 0.056 mm, respectively; *t*(8) = 2.669, *p* = 0.028; Figure 3).

Since MSCs appeared to minimize the amount of tunica intima hyperplasia during aneurysm development, Pearson's correlation was performed on tunica media and tunica intima thickness for those that developed intimal hyperplasia. The control group measurements were correlated (*r* = −0.977, *n* = 4, *p* = 0.023), but the experimental group measurements were not (*r* = 0.127, *n* = 6, *p* = 0.81).

### 3.3. Serum Outcomes

Serum samples were successfully obtained for all rabbits except for one day 15 sample from a control rabbit. All samples were stored in a -80°C freezer within 30 minutes of collection.

**Figure 4:**
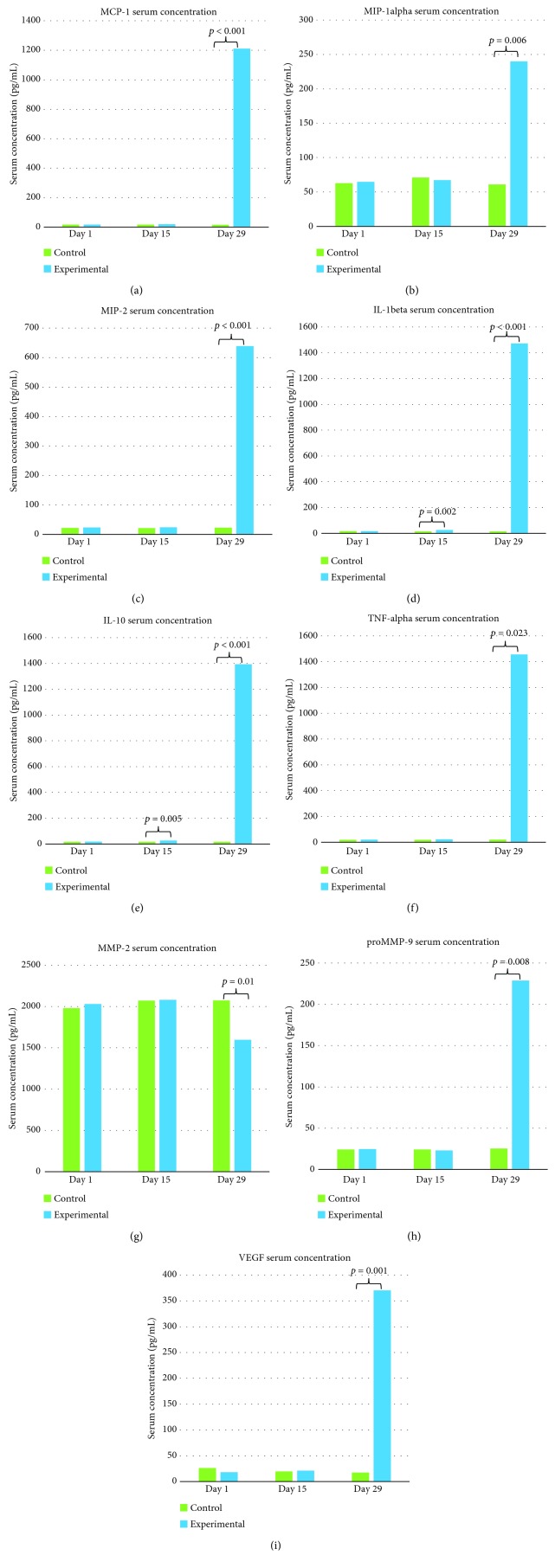
Cytokine and MMP serum concentrations in the control and experimental groups on days 1, 15, and 29 (*n* = 10). (a) MCP-1*α*, (b) MIP-1*α*, (c) MIP-2, (d) IL-1*β*, (e) IL-10, (f) TNF-*α*, (g) MMP-2, (h) proMMP-9, and (i) VEGF. Within-subject analyses revealed no statistically significant differences in the control group cytokine/MMP levels over 29 days, but a significant increase in MIP-2, IL-10, and proMMP-9 on day 29, with a significant decrease in MMP-2 on day 29. Red *p* values show significant findings on between-subject analyses.

A within-subject analysis was conducted which revealed that the control group did not demonstrate any change in serum cytokines over the course of 29 days (Figure 4). However, four of the nine measured cytokines and MMPs exhibited temporal changes in the experimental group. Friedman tests of IL-10, MIP-2, MMP-2, and proMMP-9 revealed significant differences (*χ*^2^(2) = 6.162, *p* = 0.046; *χ*^2^ [2] = 14.778, *p* = 0.001; *χ*^2^ [2] = 8.316, *p* = 0.016; and *χ*^2^ [2] = 11.545, *p* = 0.003; respectively). *Post hoc* testing for these two cytokines and two MMPs was performed using Wilcoxon Signed-Rank tests with a Bonferroni correction. IL-10 was significantly higher on day 29 than both day 1 and day 15 (*Z* = −2.804, *p* = 0.05; *Z* = −2.618, *p* = 0.009; respectively). Similarly, MIP-2 was also significantly elevated on day 29 compare to days 1 and 15 (*Z* = −2.985, *p* = 0.003; *Z* = −3.247, *p* = 0.001; respectively). MMP-2 levels significantly decreased from day 15 to day 29 (*Z* = −2.897, *p* = 0.004) while proMMP-9 levels significantly increased from day 15 to day 29 (*Z* = −2.751, *p* = 0.006).

With regard to between-subject effects, there were no differences in baseline cytokine and MMP levels between the control and experimental groups. On day 15, both IL-1*β* and IL-10 were approximately 1.6-fold higher in the experimental group (*U* = 7.5, *p* = 0.002; *U* = 11, *p* = 0.005; respectively; Figure 4). All cytokines were significantly elevated in the experimental group on day 29 (IL-1*β*: *U* < 0.001, *p* < 0.001; IL-10: *U* < 0.001, *p* < 0.001; TNF-*α*: *U* = 20, *p* = 0.023; MIP-1*α*: *U* = 13.5, *p* = 0.006; MIP-2: *U* = 2.5, *p* < 0.001; MCP-1: *U* < 0.001, *p* < 0.001; and VEGF: *U* = 4.5, *p* = 0.001). MMP-2 was significantly lower on day 29 in the experimental group, while proMMP-9 was significantly higher (*U* = 16, *p* = 0.01; *U* = 15, *p* = 0.008; respectively; Figure 4).

### 3.4. In Vitro Outcomes

**Figure 5:**
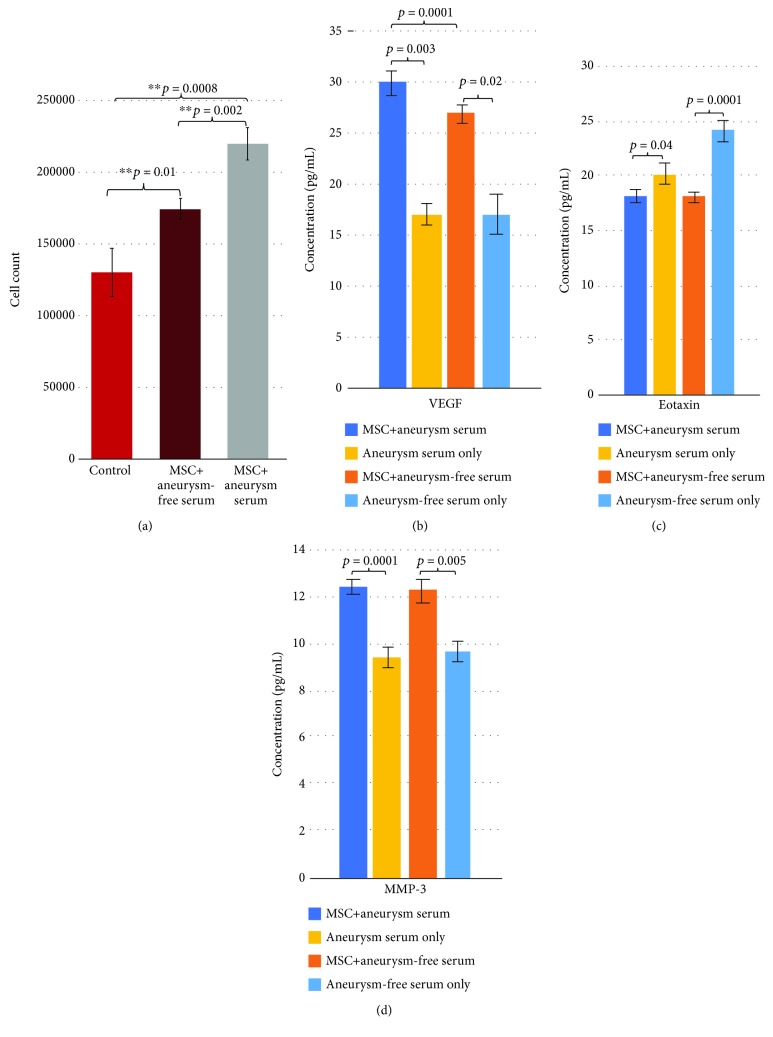
Results for the *in vitro* study. (a) Primary results from the proliferation analysis. The left bar representing MSCs grown in a serum-free environment (control), the middle group as MSCs supplemented with aneurysm-free serum, and the right being MSCs supplemented with aneurysm-containing serum. (b) A representative graph depicting trends found in group 1. (c) A representative graph showing the trends found in group 2, depicting a decrease in concentration when cultured with MSCs compared to serum-only samples. (d) Representative graph showing the trends for group 3.

For MSC proliferation, there were three groups consisting of two experimental and one control. The experimental groups were comprised of MSCs grown in media supplemented with MSC-naïve serum from animals either with or without aneurysms. The control contained MSCs grown in the absence of any serum. MSC growth was significantly heightened when introduced to serum from animals with aneurysms, as compared to the control (*p* = 0.0008). The same was also noted for MSC growth in serum from animals without aneurysms compared to the control (*p* = 0.01). Between the two experimental groups, there was also a significant increase in MSC proliferation in the presence of serum from animals with aneurysms compared to MSCs grown in media supplemented with serum from animals without aneurysms (*p* = 0.002) (Figure 5(a)).

For cytokine experiments, the experimental groups were the same as in the proliferation trials; MSCs grown in serum-free media were supplemented with serum from either aneurysm-harboring MSC-naïve animals or aneurysm-free MSC-naïve animals. The controls for this experiment were the serum samples collected from the *in vivo* experiments, to which each experimental group was compared to the corresponding serum type from which they were supplemented.

Levels of group 1 were shown to be significantly higher in the experimental MSC group that was supplemented with aneurysm-containing serum as compared to serum samples alone. Group 1 consisted of IL-9, IL-12(p40), RANTES, and VEGF, and this overall trend was depicted in Figure 5 with the results from VEGF (*p* = 0.0001, 0.001, 0.0001, and 0.003, respectively, Figure 5(b)). The levels of these cytokines were also shown to be significantly higher in the experimental MSC group that was supplemented with aneurysm-free serum as compared to serum samples alone (*p* = 0.04, 0.001, 0.0003, and 0.0001, respectively). Between the two experimental groups, IL-9, IL-12(p40), RANTES, and VEGF also showed significantly higher levels when in the presence of aneurysm-containing serum than in the presence of aneurysm-free serum (*p* = 0.005, 0.005, 0.005, and 0.0001, respectively, Supplementary Figure 1).

Group 2 showed a significant decrease in the experimental MSC group from aneurysm-containing serum supplementation compared to serum-only samples. This group consisted of Eotaxin, IL-1*β*, IL-6, LIF, MIG, MIP-1*α*, MIP-2, and proMMP-9; this overall trend was depicted in Figure 5 with the results from Eotaxin (*p* = 0.0001, 0.04, 0.0001, 0.0001, 0.0008, 0.0001, 0.0001, 0.02, and 0.01, Figure 5(c)). These cytokines also showed a significant decrease in the experimental group containing aneurysm-free serum supplementation when compared to serum-only samples (*p* = 0.04, 0.004, 0.01, 0.03, 0.0003, 0.0001, 0.0001, and 0.003, Supplementary Figure 2). Between the two experimental groups, no significant difference was found.

Group 3 consisting of G-CSF, IL-10, LIX, MMP-2, MMP-3, MMP-8, and MMP-12 showed a significant increase in the experimental MSC group from aneurysm-containing serum supplementation compared to serum-only samples; this trend was depicted in Figure 5 with the results from G-CSF (*p* = 0.04, 0.0006, 0.02, 0.04, 0.005, 0.0001, and 0.0001, Figure 5(d)). These cytokines also showed a significant increase in the experimental group containing aneurysm-free serum supplementation when compared to serum-only samples (*p* = 0.02, 0.0004, 0.0001, 0.011, 0.0001, 0.0001, and 0.0001, Supplementary Figure 3). There was no significance found between the two experimental groups.

## 4. Discussion

The primary outcome for this study was the greatest aneurysm dimension 29 days postcreation, which did not show any statistically significant difference between the MSC-exposed and control aneurysms. However, the pathogenesis of aneurysms is a complex process involving many inflammatory cascades which may have been impacted by the IV injection of MSCs. Temporal serum cytokine and MMP profiles were obtained from the arterial circulation at three time points (days 1, 15, and 29) to investigate this. The cytokine and MMP panels were selected based on their known involvement in saccular aneurysm formation, MSC physiology, and the ability to obtain antibodies with reactivity to rabbits. The resultant panel, while not exhaustive, was highly representative.

In vitro results support that MSCs exhibit both a proinflammatory and an anti-inflammatory effect. It is interesting to note that prior research has noted two subtypes of MSCs that might be responsible for this effect. MSC1, a proinflammatory MSC phenotype, is known to release MCP-1, which activates macrophages and switches them into their typical M1 proinflammatory phenotype [10, 25]. M1 macrophages in turn release MIP-1*α* and MIP-2 which continue to activate more M1 macrophages [26]. These cells secrete many cytokines, including IL-1*β*, TNF-*α*, and VEGF which, in addition to activating inflammatory cascades, also contribute to further MSC1 activation [27, 28]. They are also a source for MMP-2 and MMP-9, which have been implicated in the destruction of the internal elastic lamina [29, 30]. On the contrary, PGE2 is produced by MSC2 anti-inflammatory MSCs, which may subsequently lead to macrophage phenotype switching to an anti-inflammatory M2 cell [31, 32]. These cells in turn release cytokines such as IL-10, which has an inhibitory effect on inflammatory cascades [32]. The in vitro results support that these two subpopulations are present when introduced to an aneurysm environment. However, our experiments were not designed to elucidate the predominance of one population over another, which could be the focus for future experiments.

Baseline profiles were identical between control and experimental rabbits. On day 15, significant increases in IL-1*β* and IL-10 were noted. This trend continued to day 29. Furthermore, on day 29, some of the measured cytokines were significantly elevated in the experimental group (Figure 2). This suggests that both MSC1 and MSC2 phenotypes could be activated because both pro- and anti-inflammatory cytokines were found to be significant. This balance of antagonistic phenotypes is seen elsewhere. Specifically, stable unruptured cerebral aneurysms contain roughly equal concentrations of M1 and M2 macrophage phenotypes [33]. Ruptured aneurysms, however, have a significantly increased proportion of M1 proinflammatory macrophages residing in the wall. Therefore, it is not inconceivable that the balance of M1 and M2 macrophages, activated by MSC1 and MSC2 cells, respectively, dictates the fate of a developing aneurysm. In addition, MMP-2 and proMMP-9 decreased and increased, respectively, by day 29. As these MMPs are implicated in aneurysm formation through elastin degradation, their opposing trends provide further evidence for antagonistic phenotype activation.

MSCs are known to operate in both a paracrine and endocrine fashion [17, 34]. Assuming in this case that the injected MSCs localized to the developing aneurysm and exerted a paracrine effect on the inflammatory tissue, it is reasonable to postulate that cytokine and MMP levels in the serum would not be altered. As this was not observed, it is potentially the case that MSCs are exerting their effects in an endocrine fashion by releasing factors into the blood stream. While MSCs are not known to secrete all cytokines and MMPs measured in this experiment, this scenario is most consistent with the observations. Even if true, this does not preclude MSC localization to the developing aneurysm tissue.

An interesting finding is the drastic increase in serum cytokine and MMP variance in the experimental group on day 29. Variance was small and stable throughout the 29 days in the control group, as well as the experimental group from day 1 to day 15. While the etiology of this finding is unclear, it perhaps indicates a differential activation of MSCs. Possible reasons for this include inconsistent inflammatory signals from the developing aneurysm, variable MSC viability upon IV injection, inflammatory signals that are too weak to result in consistent MSC responses, or inconsistent cytokine immunoassays. The high variance was observed in all cytokines and MMPs, suggesting that it was likely not due to a specific inconsistent immunoassay. A small variance in experimental aneurysm size would not be expected if there was large MSC batch-to-batch viability. The reliability of the aneurysm model to produce aneurysms suggests that inconsistent inflammatory signals is likely not to blame either. Perhaps then, the inflammatory signals emitted from a very small amount of tissue are too weak to cross the MSC activation threshold when administered far from the aneurysm site. Removing some of the more extreme potential outliers does not alter the statistical results; thus, there is confidence in the findings.

Histological measurements were undertaken to try to understand the tissue-level effects that MSCs were eliciting through their pro- and anti-inflammatory modulation. It was found that the elastin content was similar in the control and experimental groups, suggesting that MSCs do not inhibit the elastase activity in this model. Tunica media thickness was not affected by MSCs either. Hyperplasia of the tunica intima was noted in approximately 50% of control and experimental rabbits. While again there was no difference in prevalence, when present, MSC-treated rabbits resulted in a significant reduction in the degree of hyperplasia. During aneurysm development, smooth muscle cells (SMCs) migrate from the tunica media into the tunica intima in response to endothelial injury [35]. They also undergo apoptosis, with the combination of the two resulting in thinning of the tunica media [36–38]. Perhaps then, MSCs are inhibiting SMC migration out of the tunica media, preferentially shuttling them into the apoptotic pathway instead. IL-1*β*, found in increased concentrations in the experimental rabbits, has been shown to increase SMC apoptosis [6]. This way, the tunica media would continue to thin, as it did in the control aneurysms, but tunica intima hyperplasia would be minimized. This is supported by the lack of correlation between tunica media and tunica intima thicknesses in the experimental rabbits, as opposed to the control aneurysms. It would appear then that perhaps, MSCs are acting in an anti-inflammatory manner, inhibiting an in situ vascular protective process. In this regard, MSCs may exacerbate a loss of structural integrity in the wall of a forming aneurysm. However, the then expected outcome of an increase in mean aneurysm size was not found, indicating either an insufficient magnitude of effect on gross aneurysm dimensions or the presence of a much more complicated process dictating aneurysm size and morphology. Either way, this is an interesting finding that requires more investigations to elucidate the underlying mechanisms and their implications.

The *in vitro* analysis allowed for a deeper understanding of how MSCs act in a simulated aneurysm environment. MSC proliferation was significantly higher when supplemented with animal blood serum. This was amplified further when the serum was from an aneurysm-harboring animal; this suggests that MSC growth is affected positively by the presence of an aneurysm.

IL-9, IL-12, and RANTES are all important factors for growing, generating, and attracting T cells [39–41]. The levels of these factors were significantly higher in the presence of MSCs than just in serum alone, indicating that the MSCs are playing a role in affecting the levels of these cytokines. The increase likely suggests that MSCs are contributing to the production of these cytokines. Levels were also significantly increased when introduced to aneurysm-containing serum suggesting that MSCs would produce more T cell proliferation factors when in the presence of an aneurysm. VEGF is fundamental in stimulating the formation of blood vessels and has also been found to stimulate differentiation in progenitor cells [42]. It was shown that VEGF levels increased when MSCs were supplemented with aneurysm-free serum compared to the serum levels alone. This result was amplified further when MSCs were supplemented with aneurysm-containing serum. The VEGF results corroborate the findings from the *in vivo* experiments and further suggest that MSCs are playing a direct role in the production of VEGF in an aneurysm environment.

Although there was no significant difference between the presence and the absence of an aneurysm, many chemoattractants and chemokines were depleted in the presence of MSCs. Eotaxin, IL-1*β*, IL-1, MIG, MIP-1*α*, and MIP-2 were found to be significantly reduced in the experimental MSC groups compared to serum-only samples. This suggests that MSCs are exhibiting their anti-inflammatory effects and deterring the involvement of the immune system by reducing the amount of proinflammatory factors in the environment. There was also a significant decrease in proMMP-9, which plays a key role in the degradation of the extracellular matrix. The decrease in IL-1*β*, proMMP-9, and MIP-1*α* is contradictory to the results found *in vivo*.

MMP-2, MMP-3, MMP-8, and MMP-12 were all found to be significantly increased in the presence of MSCs, suggesting that an increase in ECM breakdown may occur leading to remodeling of tissue by the cells [43]. G-CSF, a glycoprotein which upregulates the endogenous release of stem cells from bone marrow, was significantly increased in the presence of MSCs [44]. This was found regardless of aneurysm presence, signifying that administration of MSCs may lead to endogenously increased MSCs regardless of the presence of an aneurysm. IL-10, an anti-inflammatory cytokine, was also increased, regardless of aneurysm presence, contributing to the recognized anti-inflammatory effects of MSCs [45]. These results are contested by the results described in the *in vivo* experiments, suggesting that there is an underlying mechanism that needs to be further investigated. LIX is a chemoattractant which is also associated with the increased migration of neutrophils and has been shown to increase the lifespan of MSCs [46]. This factor was significantly increased in the MSC groups regardless of the presence of an aneurysm, when compared to serum sample controls. This suggests that the exogenous administration of MSCs may upregulate the production of LIX within the body.

It is interesting to note both the similarities and differences between the *in vivo* and *in vitro* experiments. VEGF was commonly increased in the presence of MSCs, leading to the conclusion that it is a fundamental cytokine produced by MSCs. The differences between the experiments were quite stark, and this could be due to the lack of variables within the *in vitro* study and the much more complex nature of an *in vivo* model. Although there were several conflicting results between the *in vivo* and *in vitro* experiments, one conclusion remains clear: MSCs respond to an aneurysm environment with both an anti-inflammatory and proinflammatory response. This interplay between proinflammatory and anti-inflammatory effects through MSCs has been noted in other studies [47]. MSCs are often thought to be immunosuppressive; however, these results are not always achieved, and a proinflammatory response can be induced [48, 49].

This study had several important limitations. First, a single dose of 5 × 10^6^ MSCs was used. It is possible that MSC response is dose-dependent, in which case the observed effect may have been minimized. A dose-response experiment is an important next step. A maximal safe dose of MSCs exists, as doses above this can lead to symptomatic pulmonary emboli [50]. This number is likely species-specific and needs to be determined in the rabbit model. Next, two MSC IV injections were administered during the aneurysm formative process to maximize MSC exposure to the inflammatory signals. The caveat with this design is that it rendered the results difficult to interpret in terms of identifying which injection was responsible for the observed changes. The serum cytokine and MMP changes were predominantly noted on day 29, suggesting that the day 15 injection was likely more efficacious than the day 1 injection, but this remains to be proven. Conceptually, this makes sense, as the inflammatory process likely takes several days to build to its peak intensity. The converse design would require far more animals but would be able to distinguish between the two injections. Another limitation was the serum cytokine and MMP immunoassay. Rabbit antibodies are very limited in availability, and trial runs were initially performed with antibodies designed for other animals with uncertain efficacies in rabbits. A mouse kit appeared to be the most precise and reliable, but the accuracy is unknown. Despite this, meaningful data was still obtained through relative comparisons between groups. Finally, some data was unable to be collected once the tissues were paraffin-embedded. Doing so denatured the proteins but was a necessary step in obtaining more important information such as histological measurements. Future studies investigating aneurysm tissue lysate protein levels as markers for various cellular processes would be useful.

## 5. Conclusions

This preclinical trial demonstrated that MSCs appear to take on both pro- and anti-inflammatory phenotypes upon IV injection into a rabbit elastase-induced saccular aneurysm model, and this was supported by *in vitro* studies. While no statistically significant changes in aneurysm size occurred, MSCs appear to significantly inhibit the degree of tunica intima hyperplasia through a yet undetermined mechanism. This study contributes to our understanding of MSCs in the inflammatory milieu of saccular aneurysms and paves the way for future studies to determine how best to utilize these cells in the prevention or treatment of saccular aneurysms.

## Data Availability

The data that support the findings of this study are available from the corresponding author, APM, upon reasonable request.

## Supplementary Materials

Supplementary Materials Supplementary Figure 1: all graphs for group 1 of in vitro results. Significance was found between MSC groups and serum groups as well as between MSC groups. This suggests the presence of an aneurysm affects the production of these cytokines by MSCs. Supplementary Figure 2: all graphs for group 2 of in vitro results. Significance was found between MSC groups and serum groups only. This suggests that the presence of an aneurysm does not affect MSC function in a serologic environment but that the presence of serum does affect function. Supplementary Figure 3: all graphs for group 2 of in vitro results. Significance was found between MSC groups and serum groups only. This suggests that the presence of an aneurysm does not affect MSC function in a serologic environment but that the presence of serum does increase function.
