# Supplementary material for: Mesenchymal Stem Cells Exhibit Both a Proinflammatory and Anti-Inflammatory Effect on Saccular Aneurysm Formation in a Rabbit Model
Source: Stem Cells Int. 2019 Jul 22;2019:3618217. doi: 10.1155/2019/3618217 (PMC6679866; doi:10.1155/2019/3618217)
Supplement: Supplementary Materials — Supplementary Figure 1: all graphs for group 1 of in vitro results. Significance was found between MSC groups and serum groups as well as between MSC groups. This suggests the presence of an aneurysm affects the production of these cytokines by MSCs. Supplementary Figure 2: all graphs for group 2 of in vitro results. Significance was found between MSC groups and serum groups only. This suggests that the presence of an aneurysm does not affect MSC function in a serologic environment but that the presence of serum does affect function. Supplementary Figure 3: all graphs for group 2 of in vitro results. Significance was found between MSC groups and serum groups only. This suggests that the presence of an aneurysm does not affect MSC function in a serologic environment but that the presence of serum does increase function. [file 3618217.f1.pdf]

Supplementary Figures:

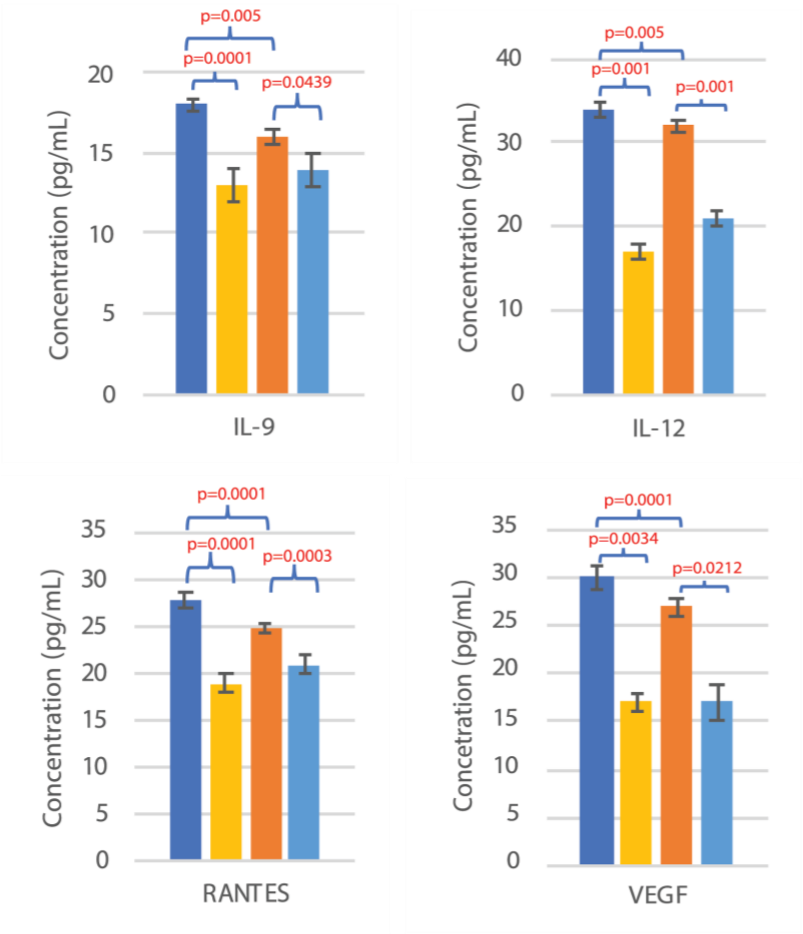

Supplementary Figure 1. All graphs for group 1 of in vitro results. Significance was found between MSC groups and serum groups as well as between MSC groups. This suggests the presence of an aneurysm affects the production of these cytokines by MSCs.

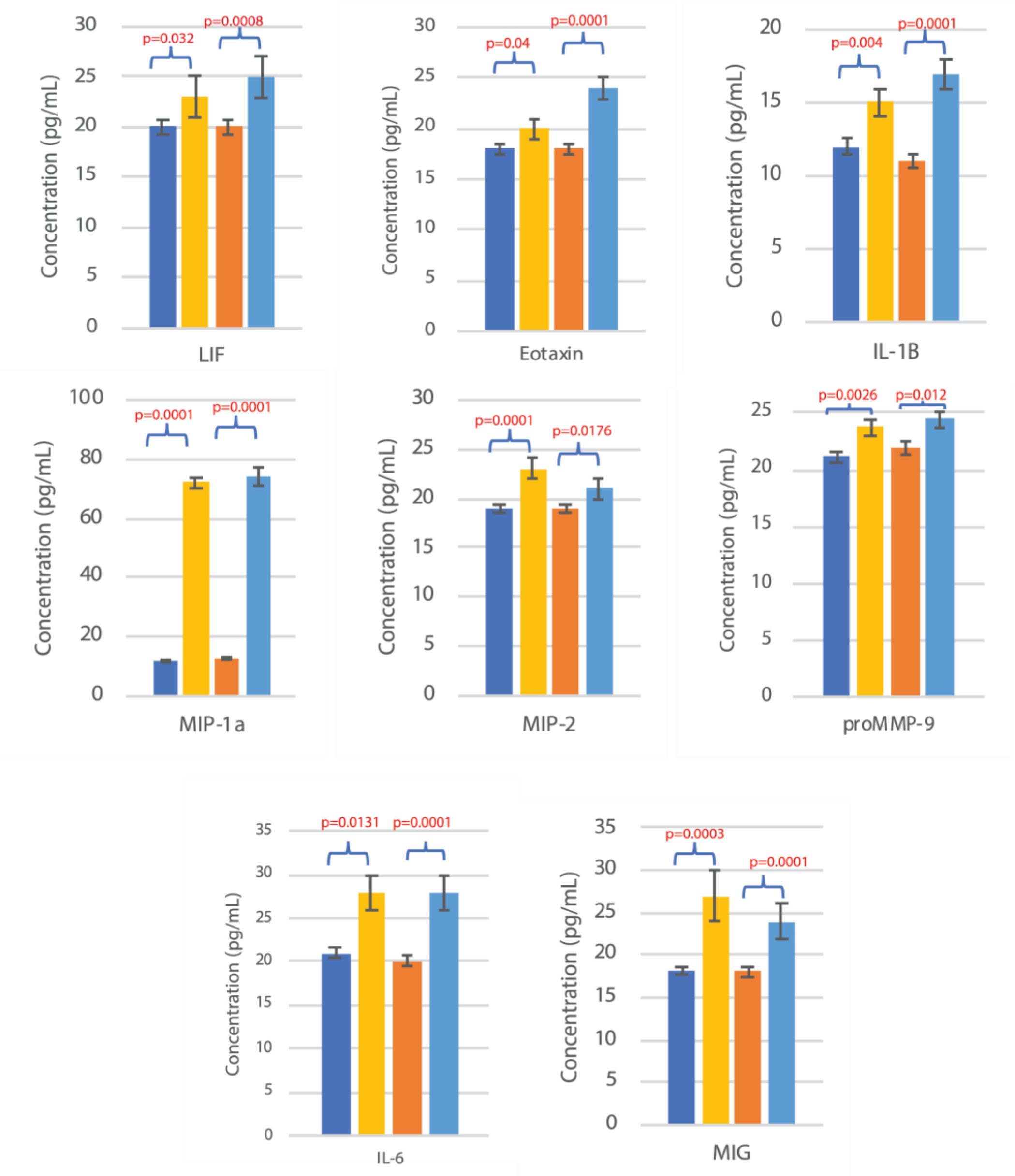

Supplementary Figure 2. All graphs for group 2 of in vitro results. Significance was found between MSC groups and serum groups only. This suggests that the presence of an aneurysm does not affect MSC function in a serologic environment, but that the presence of serum does affect function.

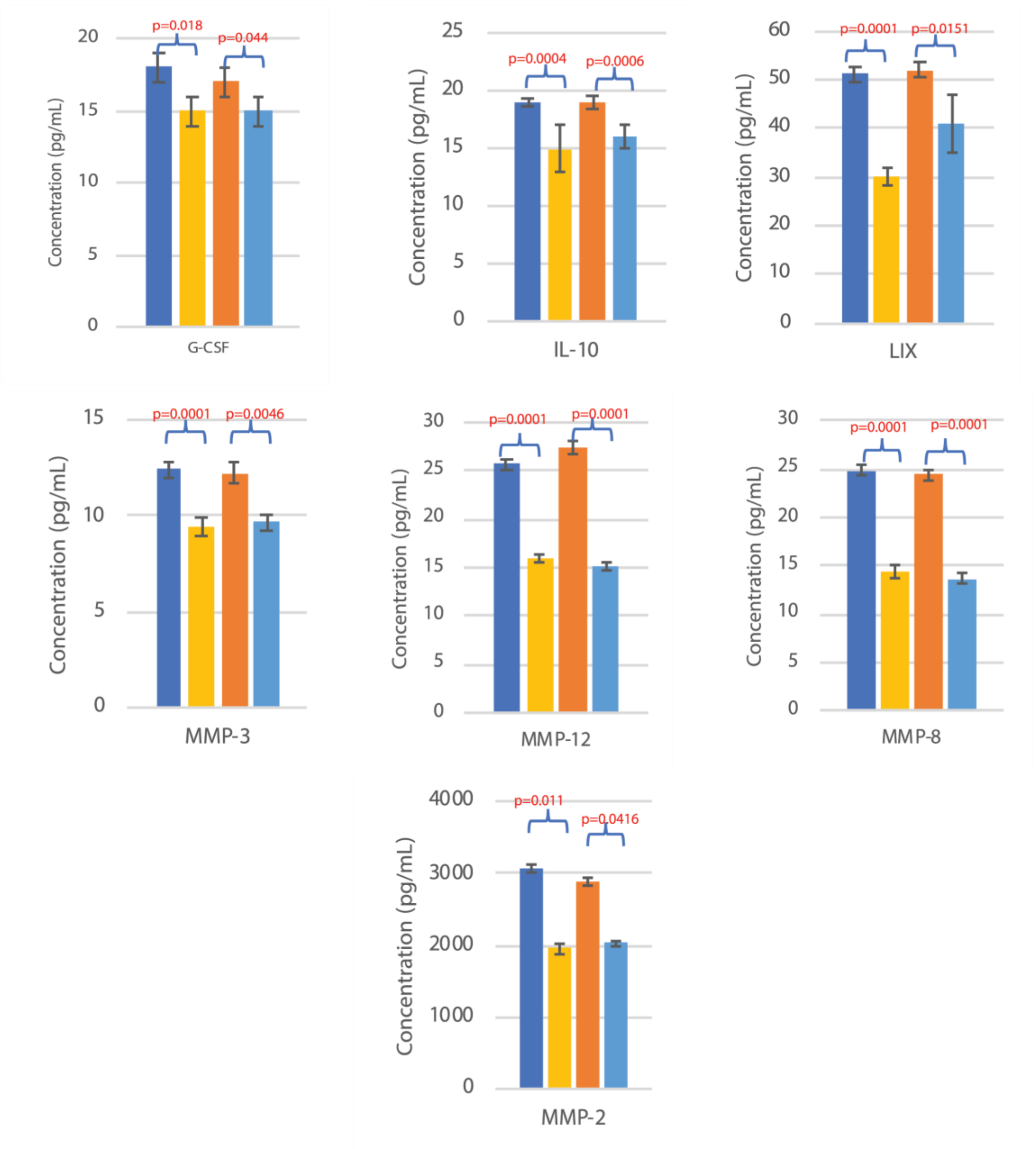

Supplementary Figure 3. All graphs for group 2 of in vitro results. Significance was found between MSC groups and serum groups only. This suggests that the presence of an aneurysm does not affect MSC function in a serologic environment, but that the presence of serum does increase function.
